# Testicular sperm results in elevated miscarriage rates compared to epididymal sperm in azoospermic patients

**DOI:** 10.1590/S1516-31802002000400007

**Published:** 2002-07-07

**Authors:** Edson Borges, Lia Mara Rossi-Ferragut, Fábio Firmbach Pasqualotto, Daniela Regina dos Santos, Cláudia Chagas Rocha, Assumpto Iaconelli

**Keywords:** Intracytoplasmic sperm injection, Azoospermia, Spermatozoa retrieval, Injeção intracitoplasmática de espermatozóide, Azoospermia, Recuperação espermática

## Abstract

**CONTEXT:**

Several sperm retrieval techniques are available for use on azoospermic men. Comparisons between spermatozoa retrieved from the testicles and epididymis in relation to pregnancy and miscarriage rates are not well established.

**OBJECTIVE:**

To compare pregnancy and miscarriage rates using sperm retrieved from the testes and epididymis using intracytoplasmic sperm injection. Furthermore, we evaluated the fertilization and pregnancy rates according to the status of the spermatozoa retrieved (motile or immotile).

**DESIGN:**

Retrospective study.

**SETTING:**

A private center for assisted fertilization.

**PARTICIPANTS:**

One hundred and eight consecutive patients who presented with azoospermia were included in our study, on whom a total of 144 retrieval procedures were performed.

**PROCEDURES:**

Of the 144 retrieval procedures, 104 were testicular sperm aspirations (TESA) and 40 were epididymal sperm aspirations (PESA). PESA was the first approach in obstructive patients (n = 68), whereas TESA was used when the former failed. For non-obstructive patients (n = 40), TESA was the method of retrieval.

**MAIN MEASUREMENTS:**

Pregnancy and miscarriage rates according to spermatozoa characteristics (motile or immotile).

**RESULTS:**

The number of cycles performed using spermatozoa retrieved from the testicles and epididymis was 81 and 30, respectively. Motile spermatozoa had higher fertilization (2PN) and pregnancy rates compared to immotile spermatozoa (p < 0.05). Also, motile spermatozoa had lower miscarriage rates compared to immotile spermatozoa (p < 0.0001). No differences were seen in pregnancy rates with testicular spermatozoa (n = 28) compared to epididymal spermatozoa (n = 13) (p = 0.1). However, the miscarriages rates were higher in spermatozoa retrieved from the testicles (n = 12) compared to epididymis retrievals (n = 1) (p = 0.01).

**CONCLUSIONS:**

Although pregnancy rates were similar when the intracytoplasmic sperm injection was performed with spermatozoa retrieved from the testicles and epididymis, the use of testicular spermatozoa yields a significantly higher miscarriage rate. It is possible that the higher miscarriage rate seen in patients using spermatozoa retrieved from the testicles is linked to high genetic sperm abnormalities.

## RESUMO

**CONTEXTO:**

Técnicas de recuperação de espermatozóides podem ser empregadas juntamente com reprodução assistida em homens azoospérmicos que desejam uma gestação. Entretanto, comparações entre recuperação de espermatozóides testiculares e epidi-dimários em relação às taxas de gestação e aborto não estão bem estabelecidas.

**OBJETIVOS:**

O objetivo deste estudo foi comparar taxas de gestação e aborto em casais submetidos à recuperação de espermatozóide testicular e epididimário associada à técnica de injeção intracitoplasmática de espermatozóide. Além disso, avaliamos as taxas de fertilização e gestação de acordo com o *status* dos espermatozóides recuperados (móveis ou imóveis).

**TIPO DE ESTUDO:**

Estudo retrospectivo.

**LOCAL:**

Centro privado de fertilização assistida.

**PARTICIPANTES:**

Cento e oito homens azoospérmicos foram incluídos em nosso estudo, totalizando 144 ciclos de recuperação espermática.

**PROCEDIMENTOS:**

Do total de procedimentos, em 104 procedeu-se à aspiração de espermatozóides testiculares e em 40 a recuperação espermática ocorreu por aspiração espermática epididimária. Esta foi o primeiro acesso em pacientes com azoospermias obstrutivas (n = 68), enquanto a aspiração de espermatozóides testiculares foi indicada nos casos de falhas prévias da aspiração espermática epididimária (nos pacientes com azoospermias obstrutivas) e em todos os casos com diagnóstico de azoospermias não- obstrutivas (n = 40).

**VARIÁVEIS ESTUDADAS:**

Taxas de gestação e aborto de acordo com a origem e características do espermatozóide (móvel ou imóvel).

**RESULTADOS:**

Oitenta e um e 30 ciclos foram realizados com espermatozóides recuperados de testículos e epidídimos, respectivamente. Espermatozóides móveis resultaram em altas taxas de fertilização (2PN) e gestação comparadas com espermatozóides imóveis (p < 0,05) e baixas taxas de aborto comparados com espermatozóides imóveis (p < 0,0001). Não foi encontrada diferença significativa na taxa de gestação com espermatozóides testiculares (n = 28) comparados com espermatozóides epididimários (n = 13) (p = 0,1). Entretanto, as taxas de aborto foram maiores com espermatozóides recuperados dos testículos (n = 12) comparados com espermatozóides epididimários (n = 1) (p = 0,01).

**CONCLUSÕES:**

Apesar das taxas de gestação serem semelhantes quando ICSI foi realizada com espermatozóides recuperados dos testículos e epidídimos, neste estudo, o uso de espermatozóide testicular produziu uma taxa de aborto significativamente maior, provavelmente devido à presença de aberrações cromossômicas nos espermatozóides.

## INTRODUCTION

Azoospermia, the absence of spermatozoa in the ejaculate, is common in the infertile male population, occurring in 10%-20% of men with abnormal semen data.^1^ Azoospermia was first recognized more than 300 years ago when Anthony van Leeuwenhoek observed in his letters to the Royal Society in 1685 that *animalcules* seen under the optical microscope in human ejaculates were responsible for conception and their absence led to infertility. These *animalcules* were later termed spermatozoa. Up until now, there has been no chance of pregnancy for couples in which the male partner had permanent azoospermia except by the use of donor insemination treatment.

The advent of intracytoplasmic sperm injection (ICSI) in 1992^2^ has enabled the treatment of male infertility conditions considered previously untreatable. These men who suffer from azoospermia are now considered suitable candidates for sperm retrieval by several different feasible techniques. Using the powerful tool of ICSI, the number of sperm necessary for a successful treatment may be limited to almost the number of oocytes obtained from the female partner. For men with nonobstructive azoospermia, retrieval of spermatozoa from the testes and its utilization in assisted reproduction techniques has provided a fertilization capacity for this sperm that is equal to that of sperm obtained from the epididymis, thereby potentially producing viable embryos and successful deliveries. Several different sperm retrieval techniques have been described with almost the same results. Spermatozoa retrieved by percutaneous epididymal sperm aspiration (PESA), testicular sperm aspiration (TESA) or open biopsy for testicular sperm extraction (TESE) can fertilize human oocytes by ICSI, leading to pregnancy.

The purpose of this study was to evaluate pregnancy and miscarriage rates in relation to men with obstructive and non-obstructive azoospermia, using TESA and PESA. Furthermore, we assessed the fertilization and pregnancy rates according to the status of the spermatozoa retrieved (motile or immotile).

## METHODS

This retrospective study was done in a private center for assisted fertilization ("Fertility", São Paulo, Brazil) from January 1995 to December 1999. It was granted approval by an authorized Ethics Committee. This investigation involved 108 consecutive men who presented with obstructive and non-obstructive azoospermia, on whom a total of 144 retrieval procedures were performed. All of these patients signed an informed consent form regarding all of the procedures, including the authorization for their results to be shown in scientific articles.

Of these procedures, 104 were TESA and 40 were PESA. Microsurgical procedures were offered for patients with obstructive azoospermia prior to the ICSI whenever possible. In the beginning, TESA was the procedure of choice in all cases, both obstructive and nonobstructive. Later on, PESA was the first approach in obstructive patients, whereas TESA was used when the former had failed.

A total of 63 patients with obstructive azoospermia were included, on whom 82 procedures were performed (42 TESA + 40 PESA). For non-obstructive patients (n = 44), TESA (n = 61) was the method of retrieval. In one patient who was unable to ejaculate, one TESA was performed. Of the total of 63 patients with obstructive azoospermia, 46 were post-vasectomy (56 procedures) and 17 (26 procedures) had other types of obstructions (8 epididymal obstructions, 1 aspermia, 7 congenital absence of the vas deferens, 1 after herniorrhaphy).

Forty-four patients (61 punctures) had clinical evaluations and laboratory data indicating non-obstructive azoospermia. A retrieval procedure was performed in one patient due to difficulties (stress) in obtaining the semen specimen. All procedures were performed on an outpatient basis, under local anesthesia in the spermatic cord. A negative pressure device was utilized with a 21-gauge butterfly needle connected to a 20 ml plastic syringe for TESA and a 27-gauge needle was employed for PESA in the same way. The needle was directed within the same puncture to various sites of the testicle and epididymis (TESA and PESA respectively), to aspirate the maximum quantity of material possible, which was transferred to a dish containing human tubal fluid (HTF/HEPES) buffer. The sample was further divided using two needles of one ml (29-gauge) and examined under 400X magnification in the microscope for the presence of sperm cells.

Discontinuous density gradients (isolation) were used in all cases to separate spermatozoa and spermatids. The gradient scales and compositions applied were three minidensity gradients containing volumes of only 0.3-0.5 ml of different density gradient materials (50%, 70% and 85% isotonic solution). After centrifugation (300 rpm), another medium bath was made up (0.3 ml) into a pellet containing spermatozoa. All metaphase II oocytes were injected for ICSI according to standard protocols.^2^ Statistical analysis using the Student *"t"* test was used for comparing the results, and p< 0.05 was considered statistically different.

## RESULTS

A total of 144 cycles was available for this study. Of these, 18 cycles were excluded, in which neither spermatozoon nor spermatids were found, and ICSI was performed using donor spermatozoa. The remaining 126 cycles had a total count of 2060 oocytes. Supplementary results are shown in Table 1.

**Table 1:** ICSI results according to the type of sperm cell injected

Variables	Immotile	Motile	ROSI + ELSI	Total
Cycles	18	94	14	126
Total number of oocytes	290	1505	265	2060
Manipulated oocytes	223	1100	144	1467
Intact oocytes	199	992	107	1298
Fertilization	127 (64%)	730 (73.6%)	49 (46%)	906 (69.8%)
Normal fertilization (2PN)	106 (53%)	605 (61%)	31 (29%)	742 (57.2%)
Embryos transferred	84	312	23	419
Pregnancy rate (%)	6 (33.3%)	37 (39.3%)	2 (14%)	45 (35.7%)
Miscarriage rate (%)	5 (83%)	10 (27%)	2 (100%)	17 (37.7%)

*No embryos were transferred in 10 cycles: six with no fertilization or morphologically abnormal embryos, and four in which all the embryos were frozen. Pregnancy was achieved in 4 cycles using donor sperm.*

Spermatozoa were successfully obtained from 112 cycles (94 motile and 18 immotile). Of 144 cycles, no spermatozoa were found in 14 of them; we found round spermatids in 13 cycles and elongated in one. In these cases, these cells were injected into oocytes (ROSI, injection of round spermatids and ELSI, using elongated spermatids).

When motile spermatozoa were injected, higher normal fertilization (2PN) was obtained and we were consequently able to note higher pregnancy rates, in comparison with immotile spermatozoa (p < 0.05). Also, the ICSI with motile spermatozoa provided lower miscarriage rates in comparison with immotile spermatozoa (p < 0.0001) (Table 1).

When sperm retrieval techniques were compared (TESA vs. PESA, or testicular and epididymal sperm), no differences in terms of pregnancy rates were noted (n = 28; n = 13 / p = 0.1, respectively). However, miscarriages rates were higher in cases that used spermatozoa retrieved from the testicles, in comparison with retrievals from the epididymis for ICSI (n = 12; n = 1/ p = 0.01, respectively) (Table 2).

**Table 2:** Pregnancy and miscarriage rates in relation to patients with testicular and epididymal sperm in azoospermic patients

Variables	TESA	PESA	P-value
Pregnancy	28 / 104 (26.9%)	13 / 40 (32.5%)	0.1
Miscarriage	12 / 204 (11.5%)	1 / 40 (2.5%)	0.01

*p < 0.05 was considered significant.*

Obstructive and non-obstructive azoospermia included, respectively, 83 and 61 ICSI cycles. Motile spermatozoa were found in 65.3% of the total number of cycles. In obstructive azoospermia, motile spermatozoa were obtained in 81.9%, against 42.6% in non-obstructive patients (p < 0.05) Table 3 shows other results from this viewpoint.

**Table 3:** Cycles divided according to kind of azoospermia and cells obtained by retrieval techniques

	Motile spermatozoa			Immotile spermatozoa		ROSI ELSI	Donor semen	Total
	TESA	PESA	TESA PESA					
Obstructive	32	27	9	9	2	2	2	83
Spermatogenesis failure	26	–	–	7	–	12	16	61
**Total**	**58**	**27**	**9**	**16**	**2**	**14**	**18**	**144**

Our results shown in Table 4 give proof that testicle spermatozoa can provide the same pregnancy rates as can epididymal spermatozoa, and higher miscarriage rates than the latter (Table 4).

**Table 4:** Pregnancy and miscarriage rates according to motility of spermatozoa

	Motile spermatozoa	Immotile spermatozoa
**Testicle**	65 cycles	16 cycles
	22 pregnancies (33.8%)	6 pregnancies (37.5%)
	7 miscarriages (10.8%)	5 miscarriages (31.2%)
**Epididymis**	29 cycles	2 cycles
	13 pregnancies (44.8%)	–
	1 miscarriage (3.4%)	–

## DISCUSSION

The introduction of ICSI has resulted in a great enhancement of fertilization and pregnancy rates in relation to patients with severely reduced sperm quality.^3^ The availability of ICSI enabled the first attempts at acetic fertilization using epididymal or testicular spermatozoa in obstructive or non-obstructive azoospermic patients, leading to viable pregnancies. According to the nature of the ICSI procedure, all natural barriers of fertilization are surpassed through direct injection of the spermatozoon into the ooplasm. Thus, the intracellular events can be initiated both through ejaculation and otherwise, allowing embryo cleavage and later development.

However, various factors affect ICSI results. These include the method of sperm retrieval, the maturity and motility status of retrieved gametes, the timing of sperm retrieval in relation to oocyte collection and the possibility of freezing the retrieved male gametes for repeated use.^4^

In our study, the procedures for sperm retrieval were efficient. Of the 144 ICSI cycles, donor semen was employed in only 18 cycles (12.5%), thus showing the real efficiency of these techniques. According to our results, the combination of ICSI with testicular or epididymal spermatozoa seems to be a solution for problems of severe male factor infertility involving azoospermia.

PESA was more efficient in cases of obstructive azoospermia than in non-obstructive ones. This fact is easily explained, because in these cases the spermatogenesis process has continued without any failure, producing healthy haploid cells (spermatozoon).

One point that needs to be analyzed is in relation to sperm motility. More motile spermatozoa (81.9% of the cases) were obtained when the etiology of azoospermia was obstructive, probably because there was no impairment of the spermatogenesis process in these cases, in comparison with non-obstructive azoospermia (42.6%).

It is known that the motility of spermatozoon used for oocyte injection is an important predictive factor for successful fertilization by ICSI.^5^^,^^6^ In some cases, no motile spermatozoa can be found in the sample. Even though fertilization and pregnancy have been reported after the injection of immotile spermatozoa, the rates are lower when compared to the injection of motile ones.^7^^,^^8^ The causes of total absence of sperm motility include the absence of dynein arms^9^ as well as abnormalities in the microtubule configurations of the sperm tail.^10^^,^^11^ Such alterations are hardly ever associated with improper centrosome function. These organelles are introduced by spermatozoa, during *in vivo* or *in vitro* fertilization, and are responsible for the formation of a zygotic centrosome and sperm aster. Sperm astral microtubules elongate throughout the cytoplasm until they come into contact with the developing female pronucleus that is translocated towards the decondensing male pronucleus, allowing single embryo formation. Abnormal cytoskeletal events during human fertilization may be providing fertilization failures and abnormal embryo development.^12^

Nevertheless, immotile sperm can be alive. In order to differentiate dead spermatozoa from viable but immotile spermatozoa, different techniques have been suggested for selecting the spermatozoa used for the ICSI procedure. Sallam et al.^13^ proposed a simple and practical method using hypo-osmotic solution, allowing for acceptable fertilization and pregnancy rates. In that work, motile spermatozoa (both epididymis and testicular) had higher normal fertilization (2PN) and pregnancy rates compared to immotile spermatozoa, leading to the conclusion that spermatozoa with better motility are essential for normal oocytic fertilization.

An important finding in that study was that, in the majority of the cases in which spermatozoa were present in the testicular biopsy, it was possible to find motile spermatozoa. Although in traditional thinking testicular spermatozoa are considered incapable of movement, some earlier studies showed that spermatozoa recovered from testes did display movement to some extent.^7^ During the TESA procedure, in addition to the epididymal capacitation, motile spermatozoa can be found in testicular samples,^5^ especially in obstructed systems. In these cases, it probably occurs because of prolonged confinement within the reproductive tract, direct contact with refluxed epididymal factors or retrograde migration of sperm after contact with the epididymal environment. Furthermore, chronic obstruction may also cause adaptation of the testicular epithelium, which allows the acquisition of intratesticular sperm motility and maturation of fertilizing capacity to occur. For long time,^14^ the success of epididymal sperm aspiration in assisted reproduction techniques has been associated with a higher proportion of motile spermatozoa.

In fact, not only is the fertilization rate influenced by sperm quality but also the embryo and blastocyst development,^15-18^ the implantation and pregnancy rates, and even spontaneous abortions,^17^ since the longest cleavage stage is linked to embryonic genomic activation.^17^^,^^19^

As well as failing to obtain differences in pregnancy rates with testicular or epididymal spermatozoa, the miscarriage rate was higher from spermatozoa retrieved from testes, in comparison to epididymis retrieval. It is probable that the use of immature testicular spermatozoa from patients with normal spermatogenesis can impair the results, i.e. involving concerns regarding genomic imprinting. While the exact timing of the imprinting of events in human gametogenesis is still unclear, some speculations have been made on the basis of indirect evidence. Ariel et al.^20^ have reported that imprinting is completed even after spermiation, and some events continue to take place during sperm transit from the epididymis.

Incidentally, since sperm quality might be affected, we can speculate that genetic etiology or damaged sperm DNA may be responsible for our results in terms of further embryo development. In fact, complex mechanisms involving epididymal transport may be beneficial for fertilization of human oocytes and embryo developmental. ^21,22^

Another point related to PESA is the risk of recovering only a few spermatozoa when compared with TESA samples. Nevertheless, in our group sufficient numbers of spermatozoa (based on oocyte numbers) were retrieved from the epididymis, providing a good number of embryos for transfer. Fortunately, emerging reproductive technologies and preliminary results have provided some hope for alternative sources of gametes, for when no spermatozoa can be found via the testicular or epididymal aspiration, with the result that these infertile patients may finally be able to conceive their own genetic child.

In this work, we used the injection of spermatozoa precursor in a few cases. Several investigators have reported pregnancies following intracytoplasmic injection of elongated (ELSI) and even round spermatids (ROSI). Pregnancies have also been reported using frozen-thawed elongated and even round spermatids. Elongated spermatids can be readily recognized morphologically, but round spermatids are very difficult to identify. This may represent another chance for the couple before semen donation; however, larger studies are needed. The rates of fertilization and pregnancy using round spermatids have been disappointing.^22,23^ Indeed, it is still unclear whether elongated or elongating spermatids provide much greater chances for success than do round spermatids. These cells cannot have the "sperm factors" responsible for oocyte activation yet. Thus, absent or abnormal oocyte activation can result in fertilization failure or in chromosomally abnormal embryos.^20-25^

We concluded that ICSI associated with sperm retrieval techniques should be employed in assisted reproduction centers, since it allows for good pregnancy rates in relation to patients with azoospermia. On the basis of our own results, in cases of obstructive azoospermia, PESA must be the first option offered to patients. If spermatozoa cannot be recovered, TESA must be offered. However, we agree with the literature with regard to female age: this technique is not a suitable solution for couples in which the female is older than 35 years of age. Since severe male factors are involved in this question, the potential risk of chromosomal abnormalities in the offspring from these men should be borne in mind, and genetic counseling (karyotyping)^21,22^ together with preimplantation genetic diagnosis should be offered to patients.

## CONCLUSION

Although pregnancy rates were similar when ICSI was performed with spermatozoa retrieved from the testicles and epididymis, the use of testicular spermatozoa yields a significantly higher miscarriage rate. Efforts should be made not only to find sperm, but also to search for the best quality possible, even if it means more time-consuming retrieval procedures, such as microsurgical sperm aspiration (MESA) for obstructive cases or microscopic guided testicular sperm extraction for non-obstructive cases. It is possible that the higher miscarriage rate seen in patients with spermatozoa retrieved from the testicles is linked to high genetic sperm abnormalities.

## References

[1] Ezeh UI (2000). Beyond the clinical classification of azoospermia: opinion. Hum Reprod.

[2] Palermo G, Joris H, Devroey P, Van Steirteghem AC (1992). Pregnancies after intracytoplasmic injection of single spermatozoon into an oocyte. Lancet.

[3] Van Steirteghem AC, Nagy ZP, Joris H (1993). High fertilization and implantation rates after intracytoplasmic sperm injection. Hum Reprod.

[4] Sallam HN, Farrag A, Agameya AF (2001). Factors affecting the success of intracytoplasmic sperm injection in cases of nonobstructive azoospermia. Reproductive Technologies.

[5] Jow WW, Steckel J, Schlegel P (1993). Motile sperm in human testis biopsy specimens. J Andrology.

[6] Moraes LAM, Clarizia A, Marinho RM, Caetano JPJ (2000). Factors of failed fertilization after intracytoplasmic sperm injection. Jornal Brasileiro de Reprodução Assistida.

[7] Nagy ZP, Joris H, Verheyen G (1998). Correlation between motility of testicular spermatozoa, testicular histology and the outcome of intracytoplasmic sperm injection. Hum Reprod.

[8] Shulman A, Feldman B, Madgar I (1999). In vitro fertilization treatment for severe male factor: the fertilization potential of immotile spermatozoa obtained by testicular extraction. Hum Reprod.

[9] Eliasson R, Mossberg B, Cammer P, Afzelius BA (1977). The immotilecilia syndrome: a congenital ciliary abnormality as an etiologic factor in chronic airway infections and male sterility. N Engl J Med.

[10] Asch R, Simerly C, Ord T (1995). The stages at which human fertilization arrests: microtubule and chromosome configurations in inseminated oocytes that failed to complete fertilization and development in humans. Hum Reprod.

[11] Hewitson LC, Simerly CR, Tengowski MW (1996). Microtubule and chromatin configurations during rhesus intracytoplasmic sperm injection: successes and failures. Biol Reprod.

[12] Hewitson LC, Simerly CR, Schatten G, Jansen R, Mortimer D Cytoplasmic endowment of organelles other than mitochondria. Towards reproductive certainty: fertility and genetics beyond 1999.

[13] Sallam HN, Farrag A, Agameya AF (2000). The use of a modified hypo-osmotic swelling test for the selection of viable ejaculated and testicular immotile spermatozoa in ICSI. Hum Reprod.

[14] Davis RO, Overstreet JW (1991). Movement characteristics of human epididymal sperm used for fertilization of human oocytes *in vitro*. Fertil Steril.

[15] Ron-El R, Herman A, Golan A (1999). Gonadotrophins and combined gonadotrophin-releasing hormone agonist –gonadotrophin protocols in a randomized prospective study. Fertil Steril.

[16] Thanki KH, Gagliardi CL, Schmidt CL (1992). Poor in vitro fertilization outcome with semen yielding low sperm density "swim-ups" is not because of altered sperm motion parameters. Fertil. Steril.

[17] Janny I, Menezo YVO (1994). Development and blastocyst formation. Mol Rep Dev.

[18] Strassburger D, Friedler S, Raziel A (2000). Very low sperm count affects the result of intracytoplasmic sperm injection. J Assist Reprod Gen.

[19] Plachot M (1996). Genetic risks associated with intracytoplasmic sperm injection. Contracept Fertil Sex.

[20] Ariel M, Cedar H, McCarrey J (1994). Developmental changes in methylation of spermatogenesis-specific genes includes reprogramming in the epididymis. Nature Genet.

[21] Yamasaki R, Farah LMS, Fragoso JB, Carrara RCV (1999). Genética e Infertilidade Masculina. I Consenso Brasileiro da Infertilidade Masculina.

[22] Almodin CG, Pereira LAC, Câmara-Minguetti VC (2000). Chromosome evaluation of infertile men prior to intracytoplasmic sperm injection. Jornal Brasileiro de Reprodução Assistida.

[23] Tesarik J, Mendoza C (1996). Spermatid injection into human oocytes. I. Laboratory techniques and special features of zygote development. Hum Reprod.

[24] Palermo GD, Schlegel PN, Hariprashad JJ (1999). Fertilization and pregnancy outcome with intracytoplasmic sperm injection for azoospermic men. Hum Reprod.

[25] Moomjy M, Sills ES, Rosenwaks Z (1998). Implication of complete fertilization failure after intracytoplasmic sperm injection for subsequent fertilization and reproductive outcome. Hum Reprod.

